# Bacterial Communities in the Fruiting Bodies and Background Soils of the White Truffle *Tuber magnatum*

**DOI:** 10.3389/fmicb.2022.864434

**Published:** 2022-05-16

**Authors:** Fabiano Sillo, Marzia Vergine, Andrea Luvisi, Alice Calvo, Gianniantonio Petruzzelli, Raffaella Balestrini, Stefano Mancuso, Luigi De Bellis, Federico Vita

**Affiliations:** ^1^National Research Council-Institute for Sustainable Plant Protection (CNR-IPSP), Turin, Italy; ^2^Department of Biological and Environmental Sciences and Technologies (DiSTeBA), University of Salento, Lecce, Italy; ^3^National Research Council, Institute of Research on Terrestrial Ecosystems (IRET), Pisa, Italy; ^4^Department of Agriculture, Food, Environment and Forestry (DAGRI), University of Florence, Florence, Italy; ^5^Department of Biology, University of Bari “Aldo Moro”, Bari, Italy

**Keywords:** metabarcoding, truffle, soil bacteria, 16S, microbiota

## Abstract

*Tuber magnatum* Picco is a greatly appreciated truffle species mainly distributed in Italy. Its price and characteristics mostly depend on its geographical origin. Truffles represent a fundamental step of the life cycle of *Tuber* species promoting spore dissemination. They consist of two main parts, gleba, the inner part, and peridium, which is in direct contact with ground soil. Within the truffle and around in the growing soil, both the occurrence and abundance of different microbial species seem to play an essential role in truffle production. The development of the next-generation sequencing (NGS) based technology has greatly improved to deepen the role of the composition of microbial communities, thus improving the knowledge of the existing relationships between microbial taxa in a specific condition. Here, we applied a metabarcoding approach to assess the differences in *T. magnatum* samples collected from three areas in Tuscany (Italy). Peridium and gleba were analyzed separately with the aim to distinguish them based on their microbial composition. Also, soil samples were collected and analyzed to compare productive and unproductive truffle grounds to confirm the presence of specific patterns linked to truffle production. Results indicate that differences occurred between truffle compartments (gleba and peridium) as well as between analyzed soils (productive and unproductive), with distinctive taxa associated. Furthermore, findings also demonstrated specific characteristics associated with truffle collection areas, thus indicating a degree of microbial selection related to different environments.

## Introduction

The ectomycorrhizal ascomycete *Tuber magnatum* Picco, known as the famous Italian white truffle, is considered the truffle species with the highest economic value due to the characteristic organoleptic properties of its edible fruiting body and its limited growing area (Riccioni et al., 2016). It mainly grows forming a mutualistic symbiosis with tree roots of plant genera such as *Quercus* spp., *Tilia* spp., *Populus* spp., *Corylus* spp., and *Salix* spp., as well as shrubs such as *Cornus sanguinea, Rosa canina, and Clematis vitalba*. This *Tuber* species is mostly collected in natural conditions in Italian and East European environments and has so far resisted the charms of domestication (Balestrini and Mello, 2015), even if recently several attempts at cultivation has proven to be successful (Bach et al., 2021). *T. magnatum* grows in marble-calcareous soils of Miocene, Pleistocene, or Holocene at a depth of 10–30 cm, sometimes even up to 80 cm (Bragato and Marjanović, 2016). It grows in 700–800 m of altitude, in areas like valley floor or moats, where the sun does not hit the ground directly. The soil should be aerated and humid, but well-drained, preferably of alluvial or sedimentary origin, and never sandy or siliceous. Minerals should not be abundant because the fungus prefers low concentrations of phosphorus and nitrogen, as well as a pH of around 7–8.5 (Mello et al., 2017).

The biology and the ecology of this species have been the objects of many scientific interests during the last 20 years (Mello et al., 2005; Bertini et al., 2006; Zampieri et al., 2014; Murat et al., 2018; Belfiori et al., 2020; Mandrile et al., 2020; Vita et al., 2020), and its life cycle was elucidated. As for other *Tuber* species (Balestrini et al., 2000; Mandrile et al., 2020), its fruiting body, namely sporocarp or ascoma, is composed of an external part ~150–350 μm in thickness, i.e., the peridium, and an inner part containing asci carrying the meiotic spores (ascospores), i.e., the gleba (Hawker, 1955). Additionally, it is also well known that bacterial communities colonize truffle ascomata (Barbieri et al., 2007, 2010; Sabella et al., 2015; Monaco et al., 2021a; Niimi et al., 2021). The role played by truffle microbiota in fruiting body maturation, aroma formation, and potential nitrogen fixation has been reported not only for *T. magnatum* but also for other truffle species, such as black truffle *Tuber melanosporum, T. borchii*, and *T. aestivum* (Frey-Klett et al., 2007; Pavić et al., 2013; Vahdatzadeh et al., 2015; Mello and Zampieri, 2017; Vita et al., 2020). It has been hypothesized that colonization of the gleba tissue by soil microbiota may occur before the differentiation of the peridium, when the primordium (a yellowish mycelial pellet) is in close contact with the soil (Antony-Babu et al., 2014). After this initial process, selected microbial species were trapped in the ascocarp during primordium development and thus protected from surrounding soil exchange by the peridium (Antony-Babu et al., 2014). A decrease in bacterial diversity in the fruiting bodies compared to the surrounding bulk soil has been observed, thus suggesting that the microbiome can be selected and excluded from ascomata colonization. Amongst all bacterial phyla identified in truffle fruiting bodies, *Proteobacteria* seems to be the most relevant, followed by Actinobacteria and Firmicutes (Antony-Babu et al., 2014; Benucci and Bonito, 2016; Niimi et al., 2021). In *Tuber* spp., *Bradyrhizobium* was found as the most abundant genus, and it has been hypothesized that it could probably be linked to the nitrogen-fixing ability typical of the Rhizobiales order (Carvalho et al., 2010; Gryndler et al., 2013). Mello et al. (2017) studied the microbial composition of the soil in areas where *T. melanosporum* fruiting bodies are found to define which species may have been subjected to selective pressure in the interactions with the fungus. Metagenomics data showed that the most abundant genera were *Actinomyces, Arthrobacter, Bacillus*, and *Flavobacterium*. The presence of *Actinobacteria* is also confirmed by other studies (Pavić et al., 2013), and *Actinobacteria* have been linked to being putatively involved in nitrogen assimilation. The analysis of microbial diversity in the fruiting body of *T. melanosporum* showed that gleba and peridium were colonized by different bacteria (Antony-Babu et al., 2014). In the peridium, more abundant phyla were *Actinobacteria, Acidobacteria*, and *Planctomycetes*, which were absent in the gleba. During the maturation process, inside the ascocarp, gleba and peridium showed a different trend in terms of bacterial communities presence: *bacteroidetes* increased in peridium and decreased in gleba, whereas *Firmicutes* remained stable in peridium and increased in gleba. *Bradyrhizobium* was found to be the dominant genus in gleba (Antony-Babu et al., 2014). Fluorescence *in situ* hybridization (FISH) analysis (Antony-Babu et al., 2014) demonstrated that the bacterial colonization specifically occurred in the gleba hyphae but not in the asci. More recently, the assessment of microbial communities in *T. indicum* showed compartmentalization of bacterial and fungal communities in different ascoma tissues, as well as a correlation with soil samples surrounding fruiting bodies and bulk soils (Liu et al., 2021). A natural selective pressure on microbes has been proposed to explain the reduction of diversity and the presence of specific taxa inside the fruiting bodies (Liu et al., 2021). *T. magnatum* fruiting bodies have a higher economic value than other truffle species, and the price may change in relation to fruiting body provenience, even at a local/regional scale. Every truffle has indeed a unique aroma, an important feature directly influencing the price of this cult food. Geographical origin is correlated to the different environmental conditions, mostly represented by temperature and humidity, that directly impact the associated microbiome and maturity stage of the truffle (Barbieri et al., 2007; Benucci and Bonito, 2016; Vita et al., 2018). Different truffle species grown in different areas were reported to be associated with different bacterial taxa (Antony-Babu et al., 2014; Splivallo et al., 2015; Niimi et al., 2021; Šiškovič et al., 2021). As for other truffle species, *T. magnatum* is colonized by a complex community of bacteria, and the soil of productive truffle sites appeared to harbor specific microbial consortia (Liu et al., 2021).

Currently, there is a rising need to understand the relationships among the *T. magnatum* ascomata-associated bacterial communities and those of the surrounding environmental bulk soil, as well as to explore the peculiar microbial diversity occurring in sites where truffles are grown and are harvested. Moreover, there is a growing need for a reliable tracking system for white truffles, and the identification of associated microbiota related to aroma compound production seems to be a promising approach. For white truffle, microbial diversity and its putative function in different compartments are less studied in comparison with other truffle species, such as *T. melanosporum* (Mello et al., 2013; Antony-Babu et al., 2014; Benucci and Bonito, 2016; Selosse et al., 2017). In another truffle species, *T. indicum* (Liu et al., 2021), it has been reported that microbial richness and diversity progressively decrease when assessing the surrounding unproductive soil, the soil of productive soils adhering to the truffle peridium, the peridium, and the gleba. This study aimed to test the hypothesis that the microbial community structure and its putative associated functions are different among *T. magnatum*-associated compartments (peridium and gleba), and among bulk soils collected in productive truffle sites and bulk soils collected outside of these sites (reported here as unproductive sites). Thus, four types of samples were considered: the peridium and the gleba of fruiting bodies, the bulk soil of productive sites, and bulk soil from unproductive sites surrounding productive truffle areas. An additional aim assessed in this study was to identify bacterial species/taxa putatively associated with the geographical area in which fruit bodies were collected and species/taxa shared between the selected sites.

## Materials and Methods

### Sampling

**Figure 1:**
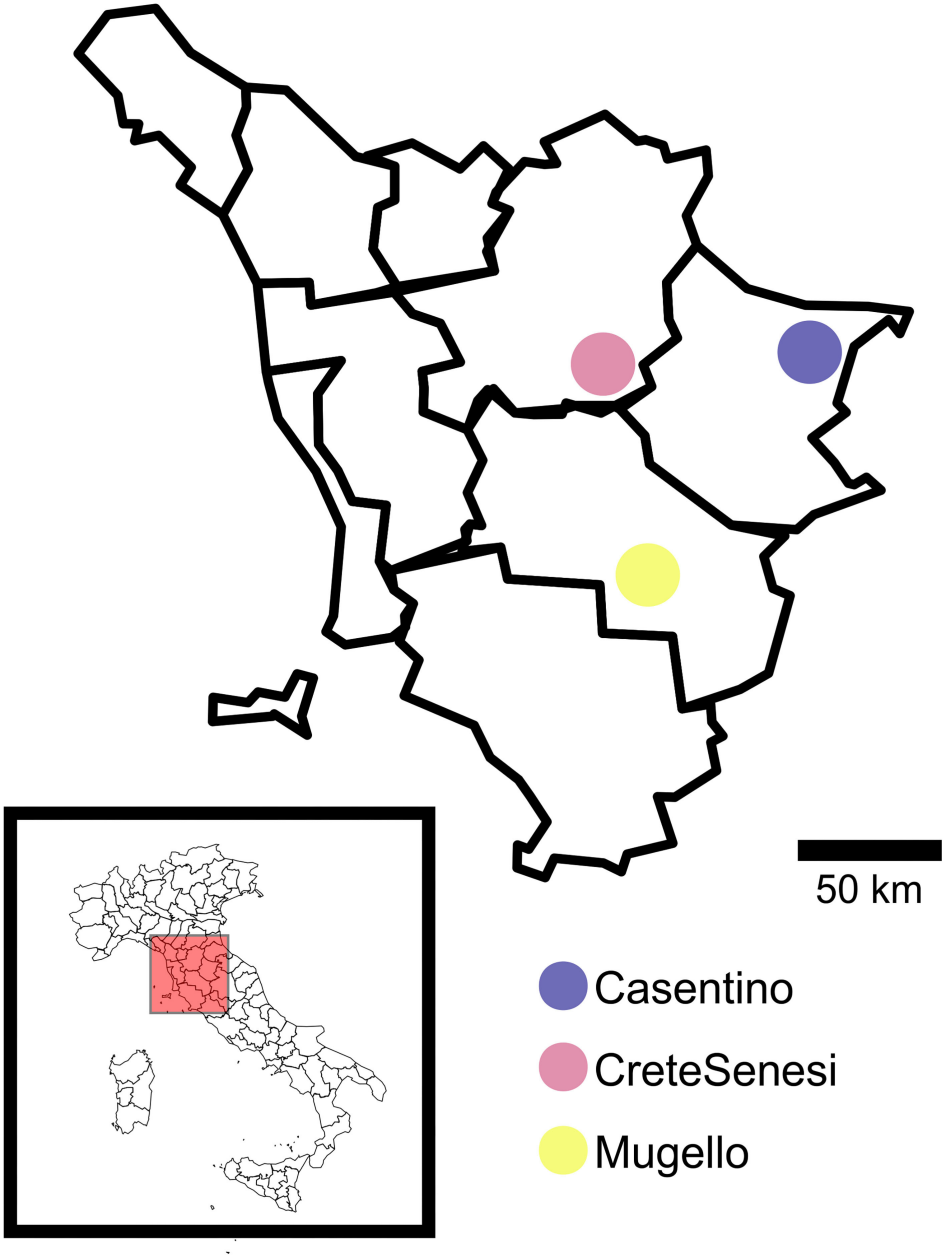
Geographical localization of truffle samples listed in Table 1. This figure was generated using R package “*maps*”.

Ascomata and soil samples were collected in three areas of Tuscany region (Italy), namely *Casentino* (Cas), *Crete Senesi* (Cre), and *Mugello* (Mug) at the end of 2018 (Figure 1). Further information on collection sites and related climatic data are reported in Tables 1, 2. Ascomata samples were represented by mature fruit bodies of *T. magnatum* collected in the three above-mentioned areas. Truffles were selected when at least 60% of the ascii containing mature spores were reached, as described in Monaco et al. (2021a). Soil samples were collected following a randomized block approach at a depth of ~10 cm, a common way of soil sampling to not compromise practicality and reproducibility (Vestergaard et al., 2017). Soil samples were composed of subsamples that represented the whole field, within an area of ~2–3 m^2^. A total of nine fruit bodies (three different fruit bodies for each of the three selected sites) and eighteen soil samples (three including “productive soils, PS” for each of the three selected sites and three including “unproductive soils, US” for each of the three selected sites). PS samples were collected from the soil surrounding the carpophores. PS and US samples were collected using the same sampling strategy mentioned above. *T. magnatum* fruiting bodies were carefully cleaned with a brush and dissected to divide the peridium (“peridium, P”) and gleba (“gleba, G”) to analyze the two compartments separately for DNA extraction. In total, 18 fungal samples (9 P and 9 G) and 18 soil samples (9 PS and 9 US) were analyzed.

**Table 1:** Sampling site and properties of sampling sites.

**Site and sample name**	**Sampling site**	**Region**	**Plants**	**Soil**	**Altitude (meters)**
Casentino (Cas)	Chiusi della Verna (AR)	Tuscany (Italy)	Turkey oak (*Quercus cerris*), hornbeam (*Carpinus Betulus*)	Arenaceous marly soil	800
Crete Senesi (Cre)	Asciano (SI)	Tuscany (Italy)	Poplar (*Populus alba*), willow (*Salix alba*)	Loose sandy loam soil	50
Mugello (Mug)	Barberino (FI)	Tuscany (Italy)	Poplar (*Populus alba*), willow (*Salix alba*)	Loose sandy loam soil	30

*Truffle samples were collected from three different sampling sites at the end of 2018*.

**Table 2:** Climatic data on the fruiting bodies sampling area for the year 2018.

		**Cas**			**Cre**			**Mug**		
**Altitude (m)**		**1,125**			**267**			**388**		
		**Max T**.	**Min T**.	**Medium T**.	**Max T**.	**Min T**.	**Medium T**.	**Max T**.	**Min T**.	**Medium T**.
Temperature (°C)	Jan	5.5	1.5	3.5	12.9	4.4	8.7	10.3	4.8	7.6
	Feb	0.5	−3.8	−1.7	8.8	0.2	4.5	5.2	0	2.6
	Mar	5	−0.1	2.5	13.1	4.3	8.7	10.2	4.1	7.2
	Apr	15.3	7.1	11.2	21.7	9.2	15.5	19.8	10.6	15.2
	May	16.9	9.6	13.3	22.9	12.4	17.7	21.7	12.9	17.3
	Jun	20.1	12.1	16.1	27.5	14.6	21.1	25.3	15.4	20.4
	Jul	24.5	15.3	19.9	31.6	17.2	24.4	29.5	17.5	23.5
	Aug	23.9	15.8	19.9	32.3	17.2	24.8	29.9	18.4	24.2
	Sep	20.6	12.6	16.6	28	14.3	21.2	26.6	15.3	21
	Oct	14.1	8.8	11.5	22.6	11.6	17.1	19.9	12.4	16.2
	Nov	8.2	4.2	6.2	15.1	7.1	11.1	12.6	7.3	10
	Dec	5.3	0.9	3.1	11.6	2.3	7	9.1	2.6	5.9
		**Monthly**	**Days**	**Cumulative**	**Monthly**	**Days**	**Cumulative**	**Monthly**	**Days**	**Cumulative**
		**rainfall**		**rainfall**	**rainfall**		**rainfall**	**rainfall**		**rainfall**
Rainfall (mm)	Jan	60.2	8	1248.4	38.2	5	900.2	83.6	15	987.2
	Feb	148.6	11		99.4	12		120.8	10	
	Mar	309.2	17		175	17		195.4	18	
	Apr	66	10		39.6	7		68.8	9	
	May	101.6	14		131.4	19		86.4	11	
	Jun	38.6	6		44.4	7		44.4	6	
	Jul	111.6	6		37.2	4		43.4	5	
	Aug	58.8	6		55.6	6		37.8	8	
	Sep	62.4	4		55	4		14.4	4	
	Oct	119.2	9		60.4	7		102.2	7	
	Nov	116.8	15		94.6	11		95.2	12	
	Dec	55.4	10		69.4	8		94.8	11	

*Data were downloaded from climate stations close to sampling sites. Cas, casentino (Chiusi della Verna; http://www.sir.toscana.it: TOS01000639); Cre, crete senesi (Asciano; http://www.sir.toscana.it; TOS03002621); Mug, mugello (Le Croci, Barberino del Mugello; http://www.sir.toscana.it: TOS01000926). Max T., maximum temperature; Min T., minimum temperature; Medium T., medium temperature*.

### DNA Extraction and Sequencing, NGS

DNA from fungal fruiting bodies was extracted as described by Edwards et al. (1991) with minor modifications. About 1 g of fungal samples was transferred into extraction bags (BIOREBA, Switzerland) and 4 ml of Tris-HCl-based extraction buffer (0.2 M of Tris–HCl pH 9, 0.4 M of LiCl, and 25 mM of EDTA) was added. Samples were then homogenized using a semi-automatic homogenizer, according to Vergine et al. (2019). The DNA solution was firstly extracted with a phenol–chloroform–isoamyl alcohol (25:24:1 ratio) solution to remove protein contaminants and then DNA was precipitated with isopropanol.

Instead, DNA from productive (productive soils, PS) and not productive (unproductive soils, US) soil samples were extracted by using NucleoSpin Soil, Mini kit for DNA (Macherey-Nagel, Germany).

The isolated DNA was then quantified and used as a template for PCR amplification with primers CS1-341F and CS2-806R targeting the V3-V4 variable regions of the 16S rDNA gene. A mixture of peptide nucleotide acid (PNA) blocker oligos (PNA Bio Inc., USA) was added for increasing accuracy in the sequencing process. Reads were collected as a couple for each sample (paired-end reads) for each condition. A total of 72 libraries were generated from the 36 starting samples. Amplicons were sequenced using an Illumina MiSeq platform (v3 chemistry) at the Génome Québec Innovation Center at the McGill University (Montréal, Canada).

In addition to morphological identification by observation of hand-made sections from the ascomata to evaluate the maturation degree (see before), molecular identification was carried out by PCR. Five out of nine samples, randomly chosen, were subjected to molecular analysis to confirm the taxonomic classification of *T. magnatum*. Briefly, DNA from samples of fruiting bodies was extracted by using DNEasy Plant Mini Kit (QIAGEN, USA), and the extracted DNA was used as a template for PCR using ITS1F/ITS4 primer pair (White et al., 1990) for molecular identification of the fungi. Amplicons were Sanger sequenced and the obtained sequences were analyzed by the blastn algorithm against NCBI nucleotide database.

### Assessment of Microbial Communities by QIIME2 Pipeline

Paired-end sequences spanning the V3–V4 regions of the bacterial 16S rRNA were analyzed using QIIME2 (Quantitative Insights Into Microbial Ecology 2) version 2020.11. Reads were imported into the QIIME2 environment and quality checking/filtering was performed. Paired-end sequences were trimmed at the first 20 bases and truncated at 200 bases from the start. Sequences were denoised through Deblur (Amir et al., 2017) embedded in QIIME2. Alignments of representative sequences were performed using the MAFFT program embedded in QIIME2, and the phylogenetic tree was generated with q2-phylogeny plugin. The *q2-feature-classifier* plugin was used to assign taxonomy to related sequences. A Naive Bayes classifier pre-trained on operational taxonomic units (99% identity) sequences on the “SILVA 138 99% OTUs full-length sequences” database was used for taxonomic assignment. Chloroplast and mitochondria sequences were removed by filtering from the resulting operational taxonomic units (OTU) table with the “filter-features” parameter of QIIME2. Bacterial community profiling by α- and β-diversity analysis was performed using the core-metrics-phylogenetic pipeline in the q2-diversity plugin within QIIME2. In particular, the diversity in the samples (α-diversity) was calculated using both Chao1 and Shannon indexes. The diversity among samples (β-diversity) was calculated by both Bray Curtis (Beals, 1984) and weighted Unifrac (Chang et al., 2011) and visualized in a two-dimensional principal coordinates analysis (PCoA).

Output from QIIME2 (feature table and related taxonomy) was converted to .*biom* file and used with microbiome analyst (Chong et al., 2020) for the visualization and statistical assessment of data. Data were filtered to remove low quality and not informative features by setting “5” as the minimum count of features (10% of prevalence in samples), as well as 10% of features with low variance in samples based on IQR. Rarefying of data was also performed to reduce bias due to different library sizes. The lowest read count of any library was chosen as the level to which all libraries were rarefied. Data scaling was based on the total sum scaling method. Statistical assessment of the difference in taxa abundance among samples at the feature-level was performed by PermANOVA (100 permutations) for α-diversity and the analysis of similarities (ANOSIM) for β-diversity, with an adjusted p-value cutoff (FDR) set at 0.05 for both the analyses (differences were considered significant for P values of 0.05 or below). A Wilcoxon rank-sum test to identify differentially abundant taxa at the order level in PS and US samples was also performed.

To infer predicted functions (i.e., oxygen requirements, metabolism, and temperature range) from metagenomic data, METAGENassist (Arndt et al., 2012) was used. Data for METAGENassist were filtered for interquartile (IQR) and normalized range (mean-centered and divided by the standard deviation of each variable). Functions were associated with identified taxa and the differences were determined by ANOVA and Tukey HSD as a post-hoc test. The 16S libraries are available at https://doi.org/10.6084/m9.figshare.19264457.v1.

### Soil Physical–Chemical Analysis

Soil samples were collected from the 10 cm layer, air-dried, and sieved through a 2-mm sieve before laboratory analysis The chemical properties of the soil were determined according to standard analytical procedures (Sparks et al., 2020). Particle size distribution (sand, silt, and clay) was determined using the pipette method (Gee and Bauder, 1986). A t-test was performed to compare pH, EC S cm^−1^, Ca, Mg, K, P, cation exchange capacity (CEC), and soil organic matter (SOM, %) in the two types of soil samples. Mantel's test was performed to investigate the relationship between the bacterial community in soil sample structure (PS and US) and soil chemical property. The Weighted UniFrac distance of the bacterial community was compared with the Euclidian distance of each mineral content. The correlations between the two distance matrices were analyzed using R software package vegan (Oksanen et al., 2011) with 1,000 permutations.

## Results

### Microbial Composition and Richness

**Figure 2:**
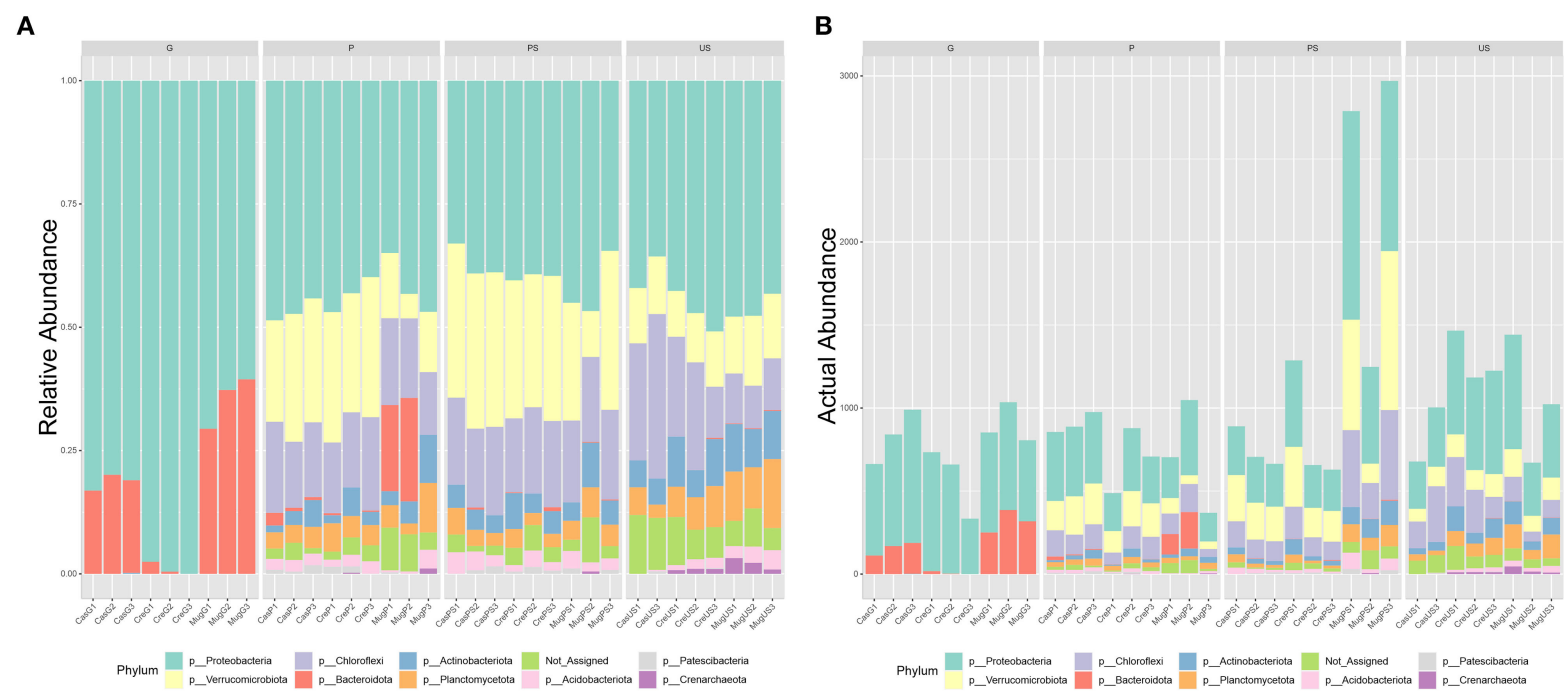
Barplots of detected OTUs at the phylum level. The percentage of detected phyla **(A)** and the actual abundance **(B)** in the four tested sample types are reported. Samples are grouped according to collection site (Cas, casentino; Cre, crete senesi; Mug, mugello) then to the truffle sample type (P, peridium; G, gleba) or the soil sample type (PS, productive soils; US, unproductive soils).

A total of 103,565 quality- and chimera-filtered bacterial sequences (considering that two libraries corresponding to sample CasUS2 were discarded because of low quality) were obtained. The average sequence counts per sample was 2,959 (total range: from 949 to 7,907). Rarefaction curves confirmed that sufficient sequencing depth was achieved for all samples (Supplementary Figure S1). The percentage of OTUs and the observed actual abundance in the four tested sample types (G, P, PS, US) are reported as taxa-stacked barplots in Figure 2, and the table of identified OTUs at different levels (phylum, class, order, family, and genus) is included as Supplementary Material (Supplementary Table S1). Sequencing results showed a massive presence of *Proteobacteria* in all samples (7,779 sequences, 44.1% of the total abundance), followed by *Chloroflexi* (2,595 sequences, 14.71%), *Verrucomicrobiota* (2,193 sequences, 12.43%), *Bacteroidota* (1,434 sequences, 8.13%), *Planctomycetota* (1,121 sequences, 6.35%), *Actinobacteriota* (766 sequences, 4.34%), and *Acidobacteriota* (629 sequences, 3.57%). In G samples, the bacterial community was mainly represented by *Proteobacteria* and *Bacteroidota*, accounting for about 98% of total microbial abundance, and *Firmicutes* were also present (1.8%). The main identified classes were *Gammaproteobacteria* and *Alphaproteobacteria* (77% and 23% respectively, of the *Proteobacteria*), while the class *Bacteroidia* was the only representative class in *Bacteriodiota*. In *Alphaproteobacteria*, the main orders were *Rhizobiales* (98%) and *Sphingomonadales* (2%); in *Gammaproteobacteria*, the orders *Burkholderiales, Enterobacterales*, and *Pseudomonadales* were the primary components (58, 27, and 15%, respectively).

Looking at the two compartments in P, *Proteobacteria, Chloroflexi*, and *Verrucomicrobiota* were the main phyla (36, 18, and 16%, respectively). *Proteobacteria* classes were *Alphaproteobacteria* and *Gammaproteobacteria* (75 and 25%, respectively). The main *Chloroflexii* classes were *Anaerolinae* and *Ktenodobacteria* (30.96 and 18.60%, respectively). Soil samples harbored a richer microbial composition than peridium and gleba samples (Figure 2B). Soil samples from PS showed an abundance of *Proteobacteria* 31.18%, *Verrucomicrobiota* 23.4%, *Chloroflexi* 21.52%, *Planctomycetota* 5.92%, *Actinobacteriota* 5.79%, and *Acidobacteriota* 4.79%. Similarly, soil samples US also showed an abundance of *Proteobacteria* 30.77%, *Chloroflexi* 22.76%, *Planctomycetota* 11.27%, *Verrucomicrobiota* 8.99%, *Actinobacteriota* 6.73%, and *Acidobacteriota* 6.15%. In both soil conditions, *Alphaproteobacteria* was more abundant than gamma *Proteobacteria*. The two conditions of soil samples differed significantly for *Verrucomicrobiota* (FDR = 2.511E^−5^), which was higher in PS than US, as well as *Planctomycetota* (FDR = 0.006) and *Crenarchaeota* (FDR = 0.013), which was higher in US than PS. Orders differentially abundant in the two soil conditions are represented in Table 3. At the genus level, *Phenylobacterium* genus was found almost exclusively in P and PS, while *Flavobacterium* genus was mainly detected in G and P samples (Supplementary Figures S2, S3).

**Table 3:** Outcomes of comparison of the bacterial composition of PS and US samples.

**Order**			**log2_median_ratio**	**Median_diff**	**Mean_diff**	***p*** **-value**
*Caulobacterales*	PS	US	Inf	0.00772	0.00761	**0.00079**
*Gemmatales*	PS	US	−2.00000	−0.02317	−0.02512	**0.00121**
*Ktedonobacterales*	PS	US	5.45943	0.04151	0.03668	**0.00193**
*Opitutales*	PS	US	1.21150	0.02413	0.02134	**0.00205**
*Sphingomonadales*	PS	US	0.42257	0.06274	0.06513	**0.00282**
*Pedosphaerales*	PS	US	1.41009	0.11680	0.10819	**0.00451**
*Saccharimonadales*	PS	US	Inf	0.00772	0.00705	**0.00573**
*Nitrososphaerales*	PS	US	–Inf	−0.04826	−0.03939	**0.01428**
*Chloroflexales*	PS	US	1.53051	0.01641	0.01389	**0.01550**
*Pirellulales*	PS	US	−1.09954	−0.01544	−0.01389	**0.01811**
*Lactobacillales*	PS	US	Inf	0.00193	0.00172	**0.01932**
*Fimbriimonadales*	PS	US	–Inf	−0.00483	−0.00445	**0.02170**
*Phycisphaerales*	PS	US	–Inf	−0.00386	−0.00464	**0.04036**
*Sphingobacteriales*	PS	US	Inf	0.00386	0.00413	**0.04457**
*Steroidobacterales*	PS	US	0.00000	0.00000	0.00257	**0.04483**
*Rokubacteriales*	PS	US	2.00000	0.00869	0.00517	**0.04621**
*Gaiellales*	PS	US	0.41504	0.00965	0.00839	**0.04689**

*Orders differentially present were reported, along with log2 median ratio between the two conditions, the median and mean difference, and the p-value of the Wilcoxon test applied to assess the significance of the difference. Significant p-value are in bold*.

### Microbial Diversity and Variation in Microbial Community

**Figure 3:**
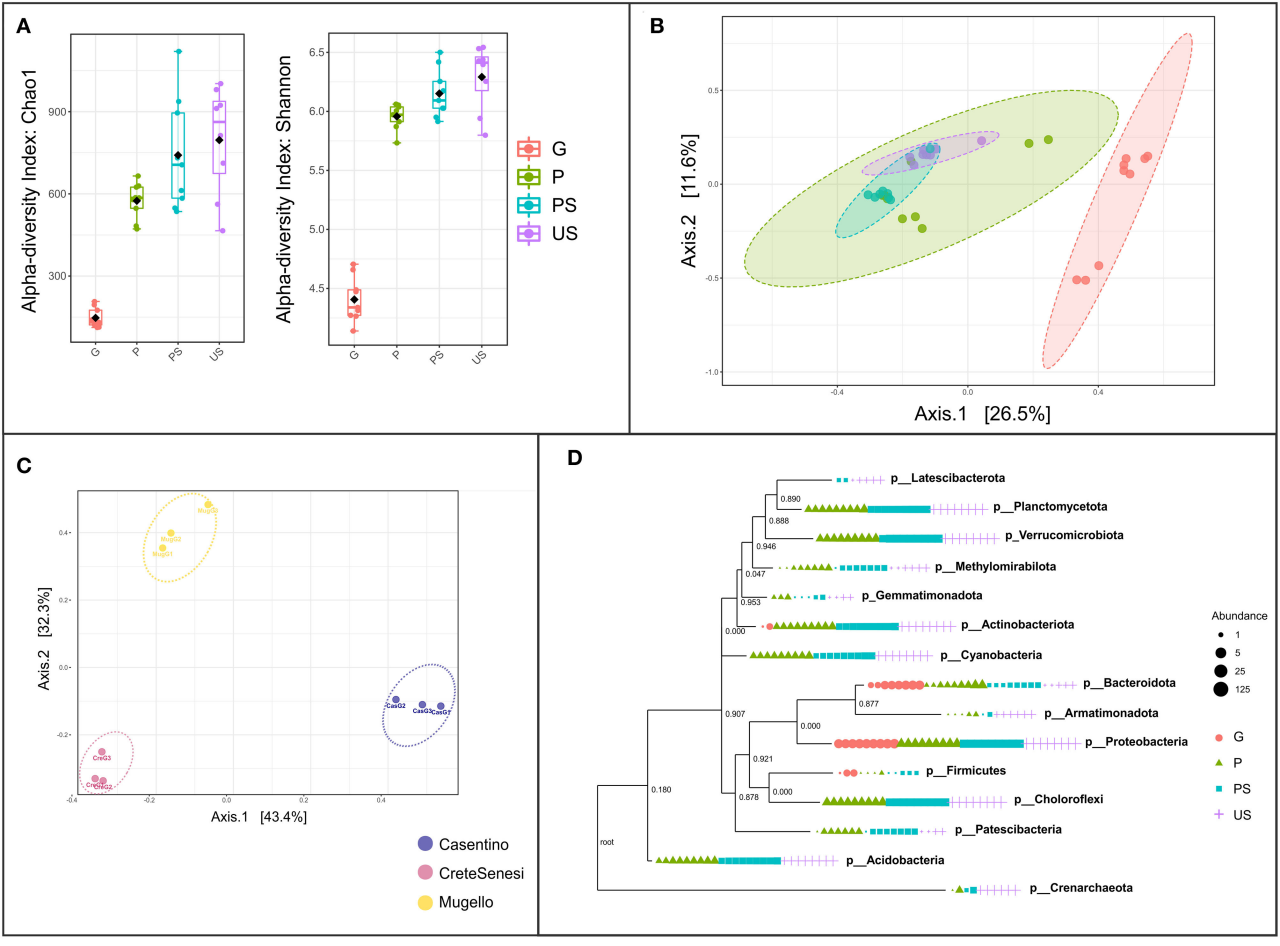
Assessment of the microbial diversity within and between samples. **(A)** Alpha-diversity based on Shannon and Chao1 indexes, at feature level; **(B)** beta-diversity based on Bray-Curtis index, at the feature level. Red, green, cyan, and purple dots represent G, P, PS, and US samples, respectively; **(C)** beta-diversity based on weighted UniFrac distance at feature level on G (gleba) samples only; **(D)** phylogenetic tree (maximum-likelihood tree via raxml). Samples are grouped according to collection site (Cas, casentino; Cre, crete senesi; Mug, mugello) then to the truffle sample type (P, peridium; G, gleba) or the soil sample type (PS, productive soils; US, unproductive soils).

Bacterial α-diversity based on Shannon and Chao1 indexes significantly differs among the four tested conditions (Figure 3A). Samples G harbored the lower level of diversity, and a trend of increase in diversity indexes can be observed, from G to P samples, to PS and US soil samples (Figure 3A). Beta-diversity on all samples confirmed that bacterial communities of G samples were significantly different from that of others (ANOSIM R: 0.55828; p-value < 0.001; Figure 3B). In addition, samples of PS were slightly separated from samples from the US, while samples of P did not form a distinct cluster (Figure 3B). Assessment of β-diversity with both Bray Curtis and weighted UniFrac distance on G samples showed that the geographic origin of the fruiting bodies has a relevant impact on differentiation among samples (Figure 3C). Phylogenetic reconstruction of detected OTUs showed that G samples include related phyla. The phylum Firmicutes was detected only in G, P, and PS samples (Figure 3D). Latescibacterota, a phylogenetically distinct phylum from Planctomycetota, was found only in PS and US (Figure 3D).

### Differences in Microbial Community Functional Categories

**Figure 4:**
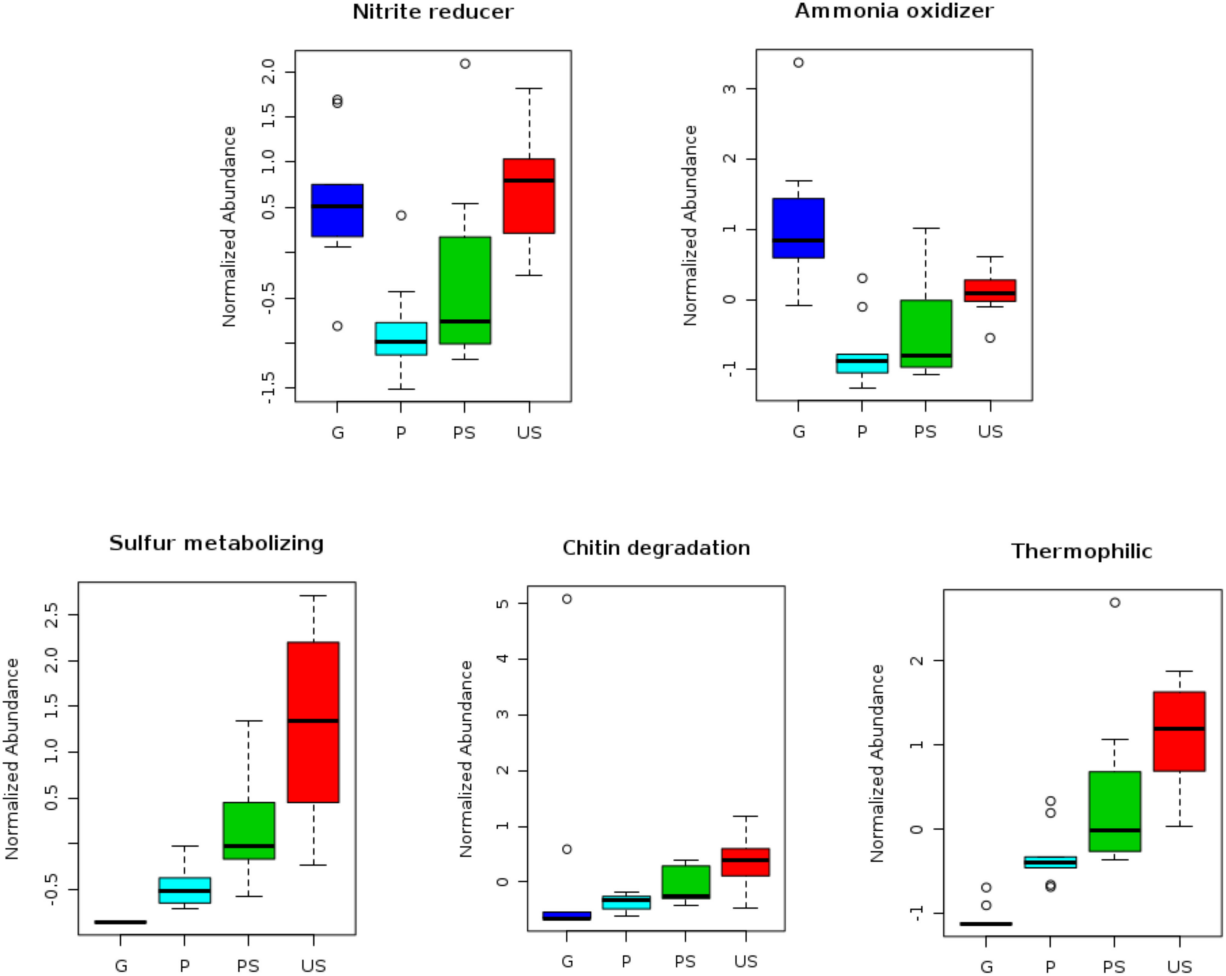
Outcomes of analyses performed with METAGENassist. Different functional categories showing significant differences among samples are shown. Blue, cyan, green, and red boxplots represent the normalized abundance of each category in G, P, PS, and US samples, respectively.

Analysis with METAGENassist allowed us to perform a taxonomic-to-phenotypic mapping based on phenotypic data covering 20 functional categories. Chitin degradation metabolism, optimal temperature range preference, sulfur and nitrogen metabolism were the functional categories showing significant differences in the four analyzed conditions (Figure 4). Particularly, chitin-degrading bacteria, as well as sulfur-metabolizing bacteria were found to decrease from US to G, with the lowest abundance in G samples and the highest in US samples (Figure 4).

Microbial taxa putatively associated with nitrite reduction process and ammonia oxidation process decrease from US to P samples, but in G samples a significant increase in abundance was observed (Figure 4). Thermophilic bacterial communities were particularly abundant in US samples, while psychrophilic taxa were mostly observed inside the fruiting bodies (G samples; Figure 4).

### Soil Physical–Chemical Features

Chemical analysis showed that both PS and US were neutral-alkaline sandy soils, with limited presence of SOM, especially in PS (Table 4). According to t-test, soil samples of PS showed high levels of Ca^2+^ compared with US samples (p < 0.05) (Supplementary Table S2). In contrast, US samples showed a higher level of SOM and higher CEC (p < 0.05) (Supplementary Table S2). No significant further differences were detected for other parameters (p > 0.05; Supplementary Table S2). Mantel test resulted in no significant association between the distance matrix from outcomes of the bacterial community in soil sample structure and soil chemical features (p = 0.460).

**Table 4:** Results of chemical analyses of PS and US samples.

**Sample** **ID**	**Geographic origins**	**pH**	**EC S cm^**−1**^**	**CEC cmol ^**(+)**^ kg^**−1**^**	**Clay %**	**Silt %**	**Sand %**	Ca g/kg^−1^^*^	Mg g/kg^−1^^*^	K g/kg^−1^^*^	P g/kg^−1^^*^	**SOM %**
PS-1	Mugello	7.94	8,300	15.8	2.1	3.6	94.3	38.32	1.3	0.43	25.1	2.52
PS-2	Crete Senesi	8.25	7,800	16.3	3.2	5.9	90.9	50.81	0.90	0.49	12.6	2.1
PS-3	Casentino	8.01	4,500	15	2.6	2.8	94.6	32.00	0.19	0.23	13.2	2.32
US-1	Mugello	8.14	8,150	17.5	1.8	2.2	96.0	30.00	0.81	0.35	22.3	3.43
US-2	Crete Senesi	8.34	1,500	18.8	3.1	1.5	95.4	22.53	0.33	0.18	15.1	3.75
US-3	Casentino	8.39	3,825	18.6	1.7	2.9	95.4	19.26	0.66	0.47	13.7	3.58

*CEC, cation exchange capacity; Ca, calcium; Mg, magnesium; K, potassium; P, phosphorus; SOM, soil organic matter*.

* *Data indicates exchangeable ion fractions*.

## Discussion

This work was dedicated to verify the bacterial communities associated with *T. magnatum* ascomata and correlate the data with the soil collected (i) at the sampling point and (ii) at an unproductive region to highlight differences related to the presence of *T. magnatum* in terms of the bacterial community. Additionally, a tissue specificity in bacterial community composition has also been evaluated, keeping separately the external region of ascomata, i.e., the peridium, and the internal one, i.e., the gleba that contains both vegetative hyphae and asci containing the spores (Balestrini et al., 2000). A decrease in alpha diversity between surrounding soil (productive soil) and the peridium, as previously reported for *T. indicum* (Liu et al., 2021), suggests that bacterial community selection occurs inside the ascomata. Outcomes of beta-diversity analysis suggest that the ascomata are composed of complex microniches, including bacteria associated with specific putative functions, i.e., nitrogen metabolism, and acclimation to sub-optimal temperatures, i.e., psychrophilic bacteria.

Data from soil chemical analysis also confirm this trend. By observing, we may find soil samples have distinct properties, leading to distinguishing between productive and unproductive soils, and their own collection site. The calcium exchangeable soil fraction represents an essential element for the growth and development of fruiting bodies (Bragato and Marjanović, 2016). High Ca^2+^ content was detected in productive soils, thus confirming its role in carpophore production. Conversely, an opposite trend could be drawn for organic matter content and CEC data, which are significantly higher in unproductive areas. Considering the soil results, we can highlight no evident differences among the three sampling areas considered, where *T. magnatum* grows and develops fruit bodies in calcareous soils with pH ranging from 6.8 to 8. However, chemical analysis of soil samples indicated that pH of unproductive soil ranged approximately from 8 to 8.4, excluding that these samples are from acidic soil. The exclusive presence of *Nitrososphearales*, an order known to be a colonizer of acidic soils (Zhao et al., 2021), in unproductive samples suggests that this bacterial taxon can be a better indicator than pH of the productive potential of a soil site. In addition, *Gemmatales* order is almost exclusively present in unproductive samples and not in productive ones. This order has been recently proposed as a bioindicator for soils with elevated phosphorus concentration (Mason et al., 2021); however, no significant difference in phosphorus was detected in soil samples from productive and unproductive soils.

Looking at the single bacteria groups, *Alphaproteobacteria*, typically a monophyletic group, and *Gammaproteobacteria*, typically paraphyletic, were differentially distributed. In gleba samples, *Gammaproteobacteria* were the most abundant bacterial class. *Gammaproteobacteria* showed a wide diversity of metabolisms, including nitrogen fixation. In other species of *Tuber* spp., *Burkholderiales, Rhizobiales*, and *Enterobacteriales*, were the most abundant orders documented inside the fruit bodies (Ye et al., 2018). Intriguingly, a previous work assessing microbial communities in *T. magnatum* fruit bodies originated in Budapest (Hungary), which showed a predominant presence of alpha-proteobacteria in gleba (Splivallo et al., 2015). Samples of fruit bodies (gleba and peridium samples) from specimens collected in Italy showed *Gammaproteobacteria* as predominant over *Alphaproteobacteria*, probably due to the different geographic origins of these truffles. In this line, the beta-diversity of the gleba samples allowed us to distinguish the nine fruiting bodies based on their local origins (*Casentino, Crete Senesi, Mugello*), suggesting that associated bacteria can be used to discriminate the origin of the precious fruiting bodies. Although this interesting result should be verified on a panel of other additional fungal samples, the analysis of specific microbiomes inside truffles appears to be a promising tool for geographic traceability, even at a local scale. Several efforts have been made in the last few years to assess the geographic origins of white truffle fruiting bodies through different novel approaches based on volatile organic compound (VOC) analysis, proteomics, transcriptomics, and metabarcoding (Vita et al., 2020; Niimi et al., 2021). The use of truffle microbiota as a tool for distinguishing the geographical origin at the local scale of *T. magnatum* samples was also reported in a recent study by Monaco et al. (2021a). At the genus level, our data indicate that *Pedobacter, Burkholderia, Pseudomonas*, and *Flavobacterium* were the dominant taxa in fungal samples. *Flavobacterium* was almost exclusively present in the *T. magnatum* fruiting bodies, both in peridium and gleba. This genus has been documented as dominant in the fruiting bodies of *T. indicum* (Deng et al., 2018) and reported in the soils surrounding ascocarps of *T. melanosporum* (Antony-Babu et al., 2014; Mello and Zampieri, 2017). On the other hand, the genus *Phenylobacterium* was present in peridium and the surrounding soil. Interestingly, it has been documented that this genus can be detected in ectomycorrhizal plant roots (Toju et al., 2019) and in production soils of *Tricholoma matsutake* (An et al., 2021), an ectomycorrhizal basidiomycete associated with plant species belonging to Pinaceae.

Diverse functions have also been correlated to a different localization. Peridium and the surrounding productive soil showed a relevant presence of thermophilic taxa, whereas bacterial colonization was mainly due to psychrophilic ones inside the ascomata. Thus, it could be hypothesized that gleba-associated bacteria were also selected for optimal temperature range. It should be considered that for *T. magnatum* ascomata, the ripening processes occur during the cold season (approximately October-December), with differences in terms of pluviometric and temperature data across the collection areas. Climatic data during our sampling confirm this trend, indicating that slight differences between *Casentino* and the other two collection areas occurred. These differences could be mostly related to the different altitudes of the *Casentino* collection site, leading to lower temperatures during the second semester of the year (June–December). Another result that is worth noting is the presence of chitin-degrading bacteria in the white truffle ascomata, particularly in the peridium. It has been proposed that ammonium release may occur during chitin degradation in the frame of fruiting bodies' maturation (Wieczorek et al., 2014). The assessment of bacterial communities in gleba samples supported the hypothesis that also bacterial taxa related to ammonium oxidation process were selected from peridium to gleba of *T. magnatum*. However, in our samples, taxa potentially involved in chitin degradation were less abundant in gleba than in peridium and soil samples, suggesting that this process was not relevant at the moment of the harvesting of fungal samples. The microbial composition of peridium and soil samples (both productive and unproductive) was similar, despite differences being detected across the collection areas. In a study by Monaco et al. (2021a), it was proposed that the peridium thickness could be involved in differentiating *T. magnatum* populations. Despite the need for further investigation, the authors assess that peridium thickness could be also a suitable parameter to distinguish samples collected from different areas (Monaco et al., 2021b). Compared to our data, the peridium thicknesses could be linked with differences detected both in terms of microbial composition and different soil properties related to collection areas.

## Conclusions

In the present study, a characterization of the profiles of the bacterial communities in *T. magnatum* fruiting body compartments and associated/non-associated bulk soils has been performed. A negative trend in bacterial diversity from unproductive soils, productive soils, peridium, and gleba of fruiting bodies has been detected. Gleba microbiota seems to have specific core taxa which allow distinguishing the fruiting bodies originating from diverse areas. The existence of a niche microbiota inside the truffle gleba reinforces the hypothesis that a strong selection on bacterial taxa, associated with specific putative functions, may occur in *T. magnatum* fruiting bodies. In soil, the presence of particular bacterial phyla and orders, i.e., phylum *Firmicutes* in productive soils, and *Gemmatales* and *Nitrososphaerales* in unproductive soils, may allow for discriminating productive from unproductive sites in the considered areas. Also, data from soil chemical analysis confirm that some parameters, including Ca^2+^ availability and pH, represent a useful starting point to select potential sites for truffle production. In conclusion, our data report a comprehensive picture of *T. magnatum* microbiota and its associated soil, thus representing a step forward in the whole comprehension of the interactions between *T. magnatum* and the linked soil microbial communities.

## Data Availability Statement

The datasets presented in this study can be found in online repositories. The names of the repository/repositories and accession number(s) can be found below: https://figshare.com/; https://doi.org/10.6084/m9.figshare.19264457.

## Author Contributions

FV and FS designed the research. MV and GP performed the DNA extraction and soil analyses, respectively. FS and AC performed the bioinformatic and statistical analyses. FS, RB, LD, and FV drafted the paper. MV, AL, GP, and SM helped revise the manuscript with all authors contributing to the discussion of the data and the writing. All authors contributed to the article and approved the submitted version.

## Funding

This project was supported by the Fondazione Caripit, Grant/Award Number: 2018.0527.

## Conflict of Interest

The authors declare that the research was conducted in the absence of any commercial or financial relationships that could be construed as a potential conflict of interest.

## Publisher's Note

All claims expressed in this article are solely those of the authors and do not necessarily represent those of their affiliated organizations, or those of the publisher, the editors and the reviewers. Any product that may be evaluated in this article, or claim that may be made by its manufacturer, is not guaranteed or endorsed by the publisher.

## Supplementary Material

Supplementary Figure S1 Rarefaction curves of samples grouped according to sample condition. The horizontal axis indicates the sequences amount getting from Illumina the number of sequences obtained through the Illumina MiSeq sequencing platform on V3–V4 regions. The vertical axis shows the number of operational taxonomic units (OTUs), at a level of 99% sequence similarity, which approximates the number of identified bacterial species.

Supplementary Figure S2 Variation in *Flavobacterium* abundance among different soil and truffle samples. Boxplots represent abundance (filtered and log-transformed count) of the taxa in the four tested conditions.

Supplementary Figure S3 Variation in *Phenylobacterium* abundance among different soil and truffle samples. Boxplots represent abundance (filtered and log-transformed count) of the taxa in the four tested conditions.

Supplementary Table S1 List of identified OTUs at different taxonomical levels.

Supplementary Table S2 Statistical results related to physical and chemical soil data.
