# Supplementary figures and images for: Bacterial Communities in the Fruiting Bodies and Background Soils of the White Truffle Tuber magnatum
Source: Front Microbiol. 2022 May 16;13:864434. doi: 10.3389/fmicb.2022.864434 (PMC9149314; doi:10.3389/fmicb.2022.864434)

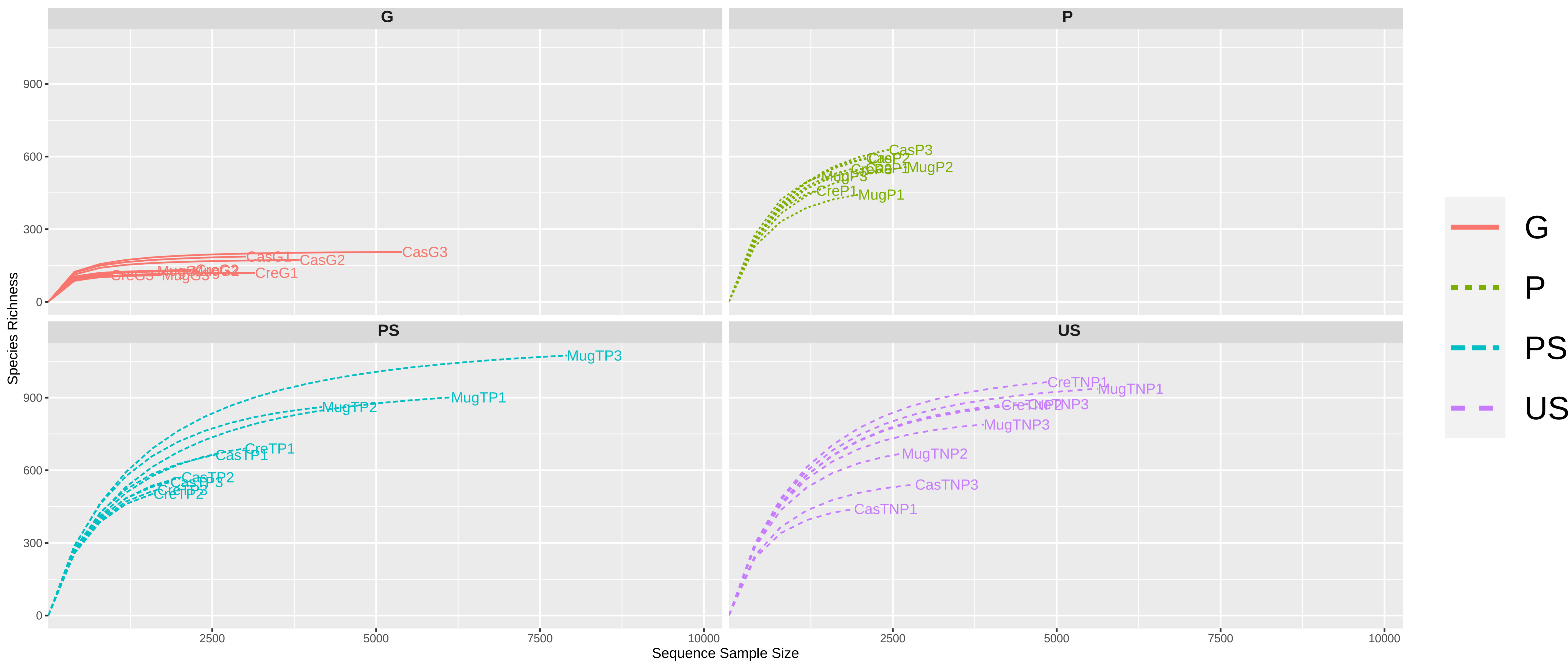

Supplement: Supplementary Figure S1 — Rarefaction curves of samples grouped according to sample condition. The horizontal axis indicates the sequences amount getting from Illumina the number of sequences obtained through the Illumina MiSeq sequencing platform on V3–V4 regions. The vertical axis shows the number of operational taxonomic units (OTUs), at a level of 99% sequence similarity, which approximates the number of identified bacterial species. [file Data_Sheet_1.PDF]

## g\_\_Flavobacterium

Filtered Count

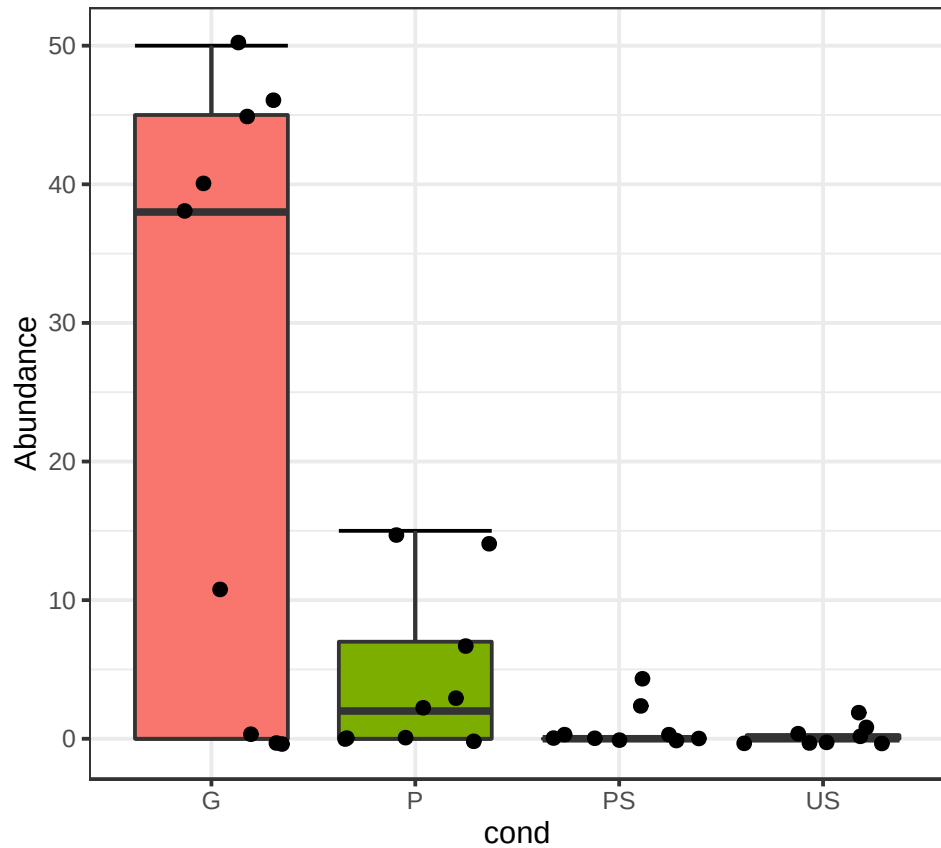

Log-transformed Count

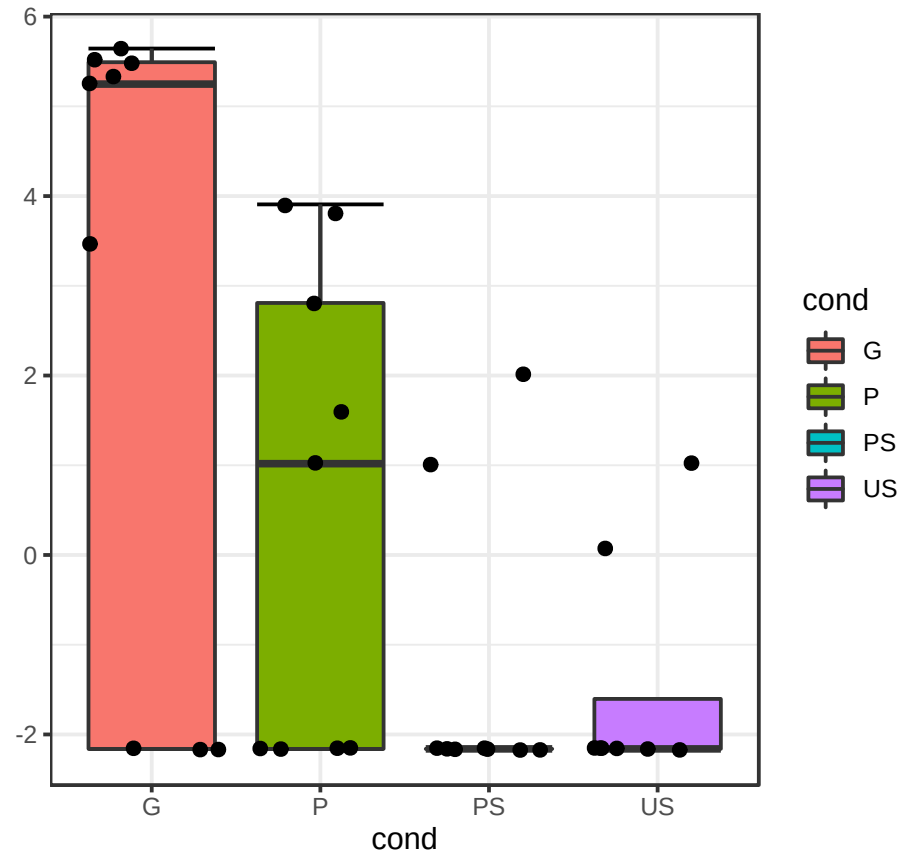

Supplement: Supplementary Figure S2 — Variation in Flavobacterium abundance among different soil and truffle samples. Boxplots represent abundance (filtered and log-transformed count) of the taxa in the four tested conditions. [file Data_Sheet_2.PDF]

## g\_\_Phenylobacterium

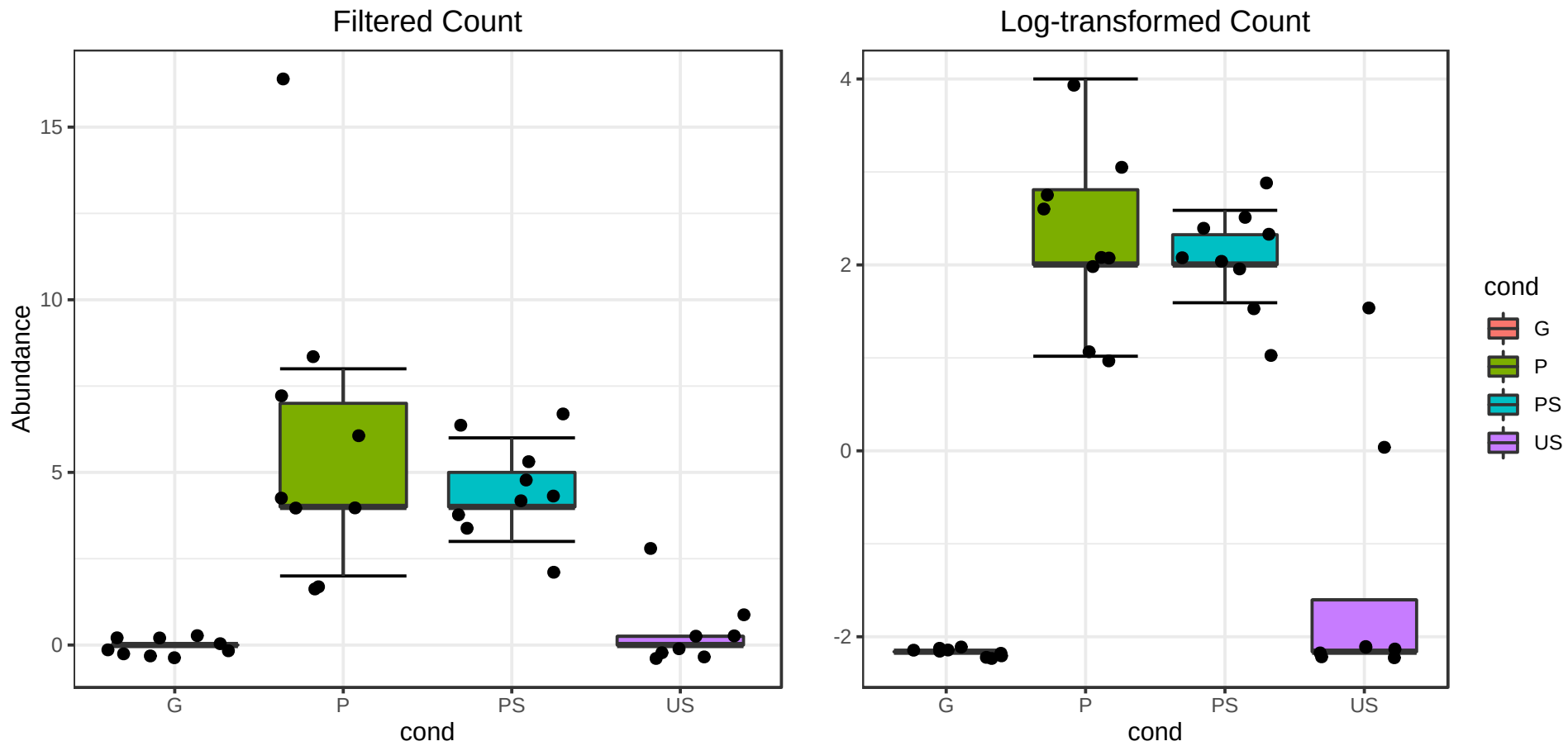

Supplement: Supplementary Figure S3 — Variation in Phenylobacterium abundance among different soil and truffle samples. Boxplots represent abundance (filtered and log-transformed count) of the taxa in the four tested conditions. [file Data_Sheet_3.PDF]
