# Supplementary material for: Bacterial Communities in the Fruiting Bodies and Background Soils of the White Truffle Tuber magnatum
Source: Front Microbiol. 2022 May 16;13:864434. doi: 10.3389/fmicb.2022.864434 (PMC9149314; doi:10.3389/fmicb.2022.864434)
Supplement: Supplementary Table S2 — Statistical results related to physical and chemical soil data. [file Table_2.DOC]

**Table S2.** Statistical assessment (t-test) on soil physical-chemical features.

|  | **PS** | **US** | **p-value t-test** |
| --- | --- | --- | --- |
| **EC S cm-1** | 6866.67±2064.78 | 4491.67±3374.75 | 0.476 |
| **CEC cmol (+) kg-1** | 15.7±0.656 | 18.3± 0.700 | 0.0093 |
| **Clay %** | 2.633± 0.551 | 2.200± 0.781 | 0.475 |
| **Silt %** | 4.100± 1.609 | 2.200± 0.700 | 0.1340 |
| **Sand %** | 93.267± 2.055 | 95.600± 0.346 | 0.1245 |
| **Ca g/kg-1*** | 40.3767± 9.3722 | 23.9300± 5.3052 | 0.0493 |
| **Mg g/kg-1*** | 0.7967± 0.5622 | 0.6000± 0.2456 | 0.6083 |
| **K g/kg-1*** | 0.3833± 0.1361 | 0.3333± 0.1457 | 0.6865 |
| **P g/kg-1*** | 16.967±7.050 | 17.033±4.614 | 0.9897 |
| **SOM %** | 2.3133± 0.2101 | 3.5867± 0.1601 | 0.0011 |
